# Bacteriophages Isolated From Turkeys Infecting Diverse *Salmonella* Serovars

**DOI:** 10.3389/fmicb.2022.933751

**Published:** 2022-07-05

**Authors:** Zhongjing Lu, John Marchant, Samantha Thompson, Henry Melgarejo, Dzhuliya Ignatova, Sandra Kopić, Rana Damaj, Hedy Trejo, Rodrigo Paramo, Ashley Reed, Fred Breidt, Sophia Kathariou

**Affiliations:** ^1^Department of Molecular and Cellular Biology, College of Science and Mathematics, Kennesaw State University, Kennesaw, GA, United States; ^2^United States Department of Agriculture, Agricultural Research Service, Washington, DC, United States; ^3^Department of Food, Bioprocessing and Nutrition Sciences, College of Agriculture and Life Sciences, North Carolina State University, Raleigh, NC, United States

**Keywords:** *Salmonella*, bacteriophage, phage ΦEnt, biocontrol, food safety

## Abstract

*Salmonella* is one of the leading causes of foodborne illnesses worldwide. The rapid emergence of multidrug-resistant *Salmonella* strains has increased global concern for salmonellosis. Recent studies have shown that bacteriophages (phages) are novel and the most promising antibacterial agents for biocontrol in foods because phages specifically kill target bacteria without affecting other bacteria, do not alter organoleptic properties or nutritional quality of foods, and are safe and environmentally friendly. Due to the vast variation in *Salmonella* serotypes, large numbers of different and highly virulent *Salmonella* phages with broad host ranges are needed. This study isolated 14 *Salmonella* phages from turkey fecal and cecal samples. Six phages (Φ205, Φ206, Φ207, ΦEnt, ΦMont, and Φ13314) were selected for characterization. These phages were from all three families in the *Caudovirales* order. Sodium dodecyl sulfate-polyacrylamide gel electrophoresis (SDS-PAGE) revealed that each phage had a unique structural protein profile. Each phage had a distinct host range. Φ207 and ΦEnt are both siphophages. They shared eight hosts, including seven different *Salmonella* serovars and one *Shigella sonnei* strain. These two phages showed different restriction banding patterns generated through *Eco*RI or *Hin*dIII digestion, but shared three bands from *Eco*RI digestion. ΦEnt displayed the broadest and very unusual host range infecting 11 *Salmonella* strains from nine serovars and three *Shigella* strains from two species, and thus was further characterized. The one-step growth curve revealed that ΦEnt had a short latent period (10 min) and relatively large burst size (100 PFU/infected cell). ΦEnt and its host showed better thermal stabilities in tryptic soy broth than in saline at 63 or 72°C. In the model food system (cucumber juice or beef broth), ΦEnt infection [regardless of the multiplicity of infections (MOIs) of 1, 10, and 100] resulted in more than 5-log_10_ reduction in *Salmonella* concentration within 4 or 5 h. Such high lytic activity combined with its remarkably broad and unusual host range and good thermal stability suggested that ΦEnt is a novel *Salmonella* phage with great potential to be used as an effective biocontrol agent against diverse *Salmonella* serovars in foods.

## Introduction

*Salmonella* is one of the leading causes of foodborne illnesses worldwide. The disease caused by *Salmonella* is known as salmonellosis, which is a type of gastroenteritis characterized by acute onset of fever, abdominal pain, diarrhea, nausea, and vomiting (WHO, 2021). Some people may develop reactive arthritis which causes joint pain, eye irritation, and painful urination. It was estimated that each year *Salmonella* causes 93.8 million cases of salmonellosis and 155,000 deaths globally (Majowicz et al., 2010). In the United States, *Salmonella* causes about 1.35 million illnesses, 26,500 hospitalizations, 420 deaths, and $365 million in direct medical costs annually (Scallan et al., 2011; Sjölund-Karlsson et al., 2013; CDC, 2021a). Natural reservoirs of *Salmonella* are domestic and wild animals, such as turkeys, chickens, cows, pigs, fish, turtles, rodents, dogs, and cats. *Salmonella* is usually transmitted to humans through contaminated foods. Many *Salmonella* outbreaks occurred in multiple states of the United States, which were often linked to animal-derived foods such as turkey products (CDC, 2021b). An increasing number of salmonellosis outbreaks were linked to fruits and vegetables, such as cucumbers, melon, sprouts, and salad greens (CDC, 2016, 2021b,2021c). The most recent multistate outbreak of salmonellosis was linked to onions, resulting in 892 illnesses and 183 hospitalizations in 38 states and Puerto Rico (CDC, 2021d).

The genus *Salmonella* includes two species: *S*. *enterica* and *S*. *bongori. S*. *enterica* is the most pathogenic species consisting of six subspecies with more than 2,600 serovars (also referred to as serotypes) (Tindall et al., 2005; Andino and Hanning, 2015). Many *S. enterica* strains are multidrug resistant and have been frequently isolated from foods in many countries (Angelika et al., 2005; Zhao et al., 2017; Zhang et al., 2018; Nikiema et al., 2021). The rapid emergence of multidrug-resistant *Salmonella* strains has increased global concern about salmonellosis, due to an increase in the mortality rate of infected patients (Gong et al., 2019; Zhan et al., 2019).

Improving food safety is a public health priority. Effective control of *Salmonella* and other bacterial pathogens is essential for food safety. Many conventional methods, such as pasteurization, chemical treatment, and irradiation, have been used in the food industries to control *Salmonella* and other bacterial pathogens. However, these methods often indiscriminately kill beneficial bacteria in foods, can adversely alter organoleptic properties and nutritional quality of foods, and are not always environmentally friendly. This has led to many attempts to develop novel alternative methods to control *Salmonella* and other foodborne bacterial pathogens in foods. Recently, bacteriophages (phages) have gained renewed interest as antimicrobial agents to reduce or eliminate specific foodborne pathogens in food products (Gordillo Altamirano and Barr, 2019; Principi et al., 2019; Picozzi et al., 2021). Phages are viruses that specifically infect and kill the target bacteria without affecting other bacteria and other organisms, such as humans (Principi et al., 2019). Phages are safe for human consumption if they are in foods and safe for use to treat bacterial infections. To date, no reports have shown that phages have any significant side effects or risks of toxicity on mammalian cells (El-Shibiny and El-Sahhar, 2017; Picozzi et al., 2021). In addition, phages do not alter the color, taste, texture, and nutritional quality of foods. Moreover, phages are natural biocontrol agents and environmentally friendly. Furthermore, the process of isolation and selection of new phages is less expensive than the development process required for antibiotics (Golkar et al., 2014; Romero-Calle et al., 2019). All these attractive features make phages novel and promising antibacterial agents. Many studies showed that lytic phages can cause rapid bacterial lysis in various foods, and they can be used as safe, natural, and effective antibacterial agents to control foodborne bacterial pathogens such as *Salmonella* (Bao et al., 2015; Kawacka et al., 2020; Vikram et al., 2020; Esmael et al., 2021; Steffan et al., 2021). Significant progress in phage research for food safety has been made toward phage applications in foods. A number of phages and their derivatives have been approved by the US Food and Drug Administration (FDA) as natural food additives to control specific foodborne pathogens. *Salmonella* phage products, such as SalmoFresh (Intralytix, Inc., Baltimore, United States), have been used in meat products to control *Salmonella*. Due to the vast variation in *Salmonella* serovars, a wide variety and a large number of highly virulent *Salmonella* phages with a broad host range are in great need. The availability of a variety of *Salmonella* phages can also be useful to help combat the possible phage resistance. In this work, we isolated and characterized *Salmonella* phages obtained from turkeys, and evaluated the potential of the phage with the broadest and very unusual host range as an effective biocontrol agent against multiple *Salmonella* serovars in cucumber juice (CJ) and beef broth (BB) as model food systems.

## Materials and Methods

### Bacterial Strains and Culture Conditions

A total of 22 *Salmonella enterica* strains from various serovars and 10 other Gram-negative bacterial strains were used in this study (Tables 1, 2). Most of these strains were from our culture collections, whereas a few strains were purchased from the American Type Culture Collection (ATCC, Gaithersburg, MD, United States). All bacterial strains were maintained at –80°C in tryptic soy broth (TSB) supplemented with 20% glycerol (v/v). Working stocks were maintained on tryptic soy agar (TSA) at 4°C for 1 month. Fresh overnight cultures were prepared by inoculating 10 ml of TSB with an isolated colony from a TSA plate and incubated at 37°C statically for 12 h. Bacterial concentration was measured by the plate count method using TSA plates.

**TABLE 1:** *Salmonella enterica* serovars and strains used for phage isolation and phages isolated in this study.

*Salmonella*		Phage	
ID*^a^*	Strain	Name	Source
B0205	*S.* Belem	**Φ205** *^b^*	Tc*^c^*
		Φ205-2	Tf*^d^*
B0206	*S.* Branderup	**Φ206**	Tc
B0207	*S.* Cerro	**Φ207**	Tc
B0208	*S.* Enteritidis	Φ208	Tc
		Φ208-2	Tf
B0209	*S.* Newport	Φ209	Tc
		Φ209-2	Tf
B0210	*S.* Typhimurium	Φ210	Tc
125109	*S.* Enteridis	**ΦEnt**	Tf
		ΦEnt-2	Tc
SGSC4911	*S.* Newport	ΦNew	Tc
SARB 30	*S.* Montevideo	**ΦMont**	Tf
ATCC 13314	*S*. ssp. *arizonae*	**Φ13314**	Tc
B0038	S. Typhimurium	N/A*^e^*	Tc or Tf
ATCC 14028	*S.* ssp. *enterica* Typhimurium	N/A	Tc or Tf

*^a^ID, Identification number of bacterium in our culture collections.*

*^b^Phages in bold were selected for characterization.*

*^c^Tc, Turkey ceca.*

*^d^Tf, Turkey feces.*

*^e^N/A, no phage isolated against the target bacterium.*

**TABLE 2:** Host ranges of six *Salmonella* phages isolated from turkeys.

Bacterial strain		Phage					
ID*^a^*	Name	Φ205	Φ206	Φ207	ΦEnt	ΦMont	Φ13314
**B0038*^b^***	*Salmonella* Typhimurium						
**B0205**	*Salmonella* Belem	**_+_ *^c^***		**+**	**+**		
**B0206**	*Salmonella* Branderup		**+**	**+**			
**B0207**	*Salmonella* Cerro	**+**		**+**	**+**		
**B0208**	*Salmonella* Enteritidis	**+**			**+**		
**B0209**	*Salmonella* Newport						
**B0210**	*Salmonella* Typhimurium				**+**		
**125109**	*Salmonella* Enteritidis	**+**		**+**	**+**		
**SGSC4911**	*Salmonella* Newport						
**SARB 30**	*Salmonella* Montevideo					**+**	
**ATCC 14028**	*Salmonella* ssp. *enterica* Typhimurium				**+**		
**ATCC 13314**	*Salmonella* ssp. *arizonae*						**+**
SGSC4914	*Salmonella* Kentucky			**+**	**+**		
SARB 59	*Salmonella* Senftenberg						
SGSC4905	*Salmonella* Infantis			**+**	**+**		
SGSC4915	*Salmonella* Heidelberg						
SARB 32	*Salmonella* Muenchen						
SGSC4906	*Salmonella* Hadar				**+**		
SARB 62	*Salmonella* Thompson			**+**	**+**	**+**	
SGSC4964	*Salmonella* Weltevreden						
S-500	*Salmonella* Braenderup			**+**	**+**		
SGSC4919	*Salmonella* Schwarzengrund	**+**					
ATCC 29903	*Shigella flexneri* 2a						
ATCC 12022	*Shigella flexneri* 2b						
ATCC 13313	*Shigella dysenteriae*				**+**		
ATCC 25931	*Shigella sonnei*				**+**		
ATCC 29930	*Shigella sonnei* (type strain)	**+**		**+**	**+**		
ATCC 9290	*Shigella sonnei*						
ATCC 8700	*Shigella boydii* 2						
B0241	*Escherichia coli* O157:H7						
ATCC 25922	*Escherichia coli*						
ATCC 13047	*Enterobacter cloacae*						

*^a^ID, Identification number of bacterium in our culture collections.*

*^b^The strains with bolded ID were used for initial phage isolation.*

*^c^+, Bacterial strain susceptible to phage infection.*

### Phage Propagation and Plaque Assay

Phages were propagated on their hosts at 37°C in TSB or on TSA unless otherwise specified. Phage titer was measured by double agar overlay plaque assay (Adams, 1959; Kropinski et al., 2009). Soft TSA (as top agar) was prepared with TSB supplemented with 0.5% agar. For plaque assay, 100 μl of overnight bacterial culture and 100 μl of phage sample were added into 3 ml of molten soft TSA maintained at 50°C. The resulting mixture was then poured onto a TSA plate. All plates were incubated overnight at 37°C before the examination.

### Model Food Systems

Cucumber juice and beef broth were used as model food systems to represent vegetable and meat products in this study. To prepare CJ, fresh cucumbers (size 3A, 44–51 mm in diameter) were washed, cut into pieces, and blended at maximum speed for 1 min (Waring Co., Torrington, CT, United States). The resulting cucumber slurry was frozen overnight. After thawing, the slurry was centrifuged at 10,000 × *g* and 4°C for 30 min. The supernatant was filtered using a bottle-top filter with a pore size of 0.45 μm. The beef broth was purchased from a local grocery store and centrifuged at 10,000 × *g* and 4°C for 1 h. The resulting supernatant was filtered using a bottle-top filter (0.45 μm pore size). Both filtered CJ and BB were aliquoted and stored at –80°C until use.

### Sample Collection and Processing

*Salmonella* phages were isolated from turkey ceca and feces. Whole ceca from 72 Hybrid tom turkeys were collected by the veterinarians of a turkey production company and were shipped overnight on ice to our lab at North Carolina State University (NCSU), Raleigh, NC, United States. Fecal materials from 10 turkeys were collected from a poultry field lab at NCSU. The cecal and fecal samples of the turkey were placed immediately in an ice box and transported to the laboratory, where they were temporarily stored in a refrigerator until processed. The contents from 72 ceca were collected and pooled together. The fecal materials from 10 turkeys were also pooled together. The pooled cecal content and pooled fecal material were separately diluted 10-fold with TSB and then centrifuged at 10,000 × *g* for 1 h. The resulting supernatants were filtered through Nalgene filtration units with 0.45 μm pore size. The two filtrates (one from ceca and one from feces) were stored at 4°C until used as potential phage sources for phage isolation.

### Phage Enrichment and Detection

Twelve *Salmonella* strains (Table 1) were used as potential target hosts for phage isolation. The phages were enriched by inoculation of 10 ml of a filtrate with 500 μl of an overnight *Salmonella* culture (approximately 1 × 10^9^ CFU/mL) and incubation at 37°C for 6 h. After incubation, 1 ml of each potentially enriched phage lysate was centrifuged at 16,110 × *g* (13,200 rpm, 5415D Benchtop Microcentrifuge, Eppendorf, United States) for 1 min. The supernatant was filtered through a syringe filter (0.45 μm pore size). The resulting filtrates were then screened for the presence of phage activity by the spot test method (Chopin et al., 1976). Briefly, 10 μl of a filtrate was spotted onto a double agar overlay plate with the top layer seeded with 100 μl of a fresh *Salmonella* culture. The spot test plates were left to dry on the bench for 20 min and then incubated overnight at 37°C. The plates were examined for phage activity indicated by the presence of a clear zone or plaques.

### Phage Isolation, Purification, and Concentration

The plates containing a clear zone or plaques from spot tests were used for initial phage isolation. Briefly, the lysis zone or plaques from a spot test plate were picked with a truncated sterile pipette tip and transferred into a tube containing 1 ml of TSB and 100 μl of an overnight target *Salmonella* culture (the same strain used for enrichment). The tube was incubated at 37°C for about 6 h. The phage lysate was centrifuged at 16,110 × *g* for 1 min to precipitate bacterial cells. The supernatant was used for plaque assay. The plaque assay plates were incubated at 37°C for 6–12 h. A well-isolated plaque was picked for another round of plaque purification. Each isolated phage underwent multiple (3–5) rounds of plaque purification.

High-titer phage lysate was prepared through the propagation of purified phage on its host in TSB at 37°C for 12 h. The phage lysate was then centrifuged at 5,000 × *g* for 30 min. The supernatant was filtered through a bottle-top filter (0.45 μm pore size). The filtered high-titer phage stock (typically ca. in the range from 10^8^ to 10^10^ PFU/ml) was stored at 4°C until use. Frozen phage stock was prepared with TSB supplemented with 20% glycerol (v/v) and stored at –80°C. A portion of high-titer phage stock was further purified and concentrated by using the methods described by Lu et al. (2003). Briefly, the filtered phage stock was treated with DNase I and RNase A, and then concentrated by polyethylene glycol precipitation. The phage pellet was resuspended in a small amount of Tris-EDTA (TE) buffer (pH 7.6). The resuspended phage was layered on a CsCl step density gradient (1.4, 1.5, and 1.7 g/ml) and ultracentrifuged at 600,000 × *g* for 6 h at 4°C. The phage bands were extracted and desalted through dialysis against 10 mM Tris buffer. The ultracentrifuge-purified high-titer (∼10^10^–10^12^ PFU/mL) phages were used for electron microscopy analysis, sodium dodecyl sulfate-polyacrylamide gel electrophoresis (SDS-PAGE), and phage DNA extraction.

### Host Range

The host range of each selected phage was determined against a panel of 22 *Salmonella* strains and 10 other Gram-negative bacterial strains (Table 2) using spot tests as described previously. In these tests, a high-titer phage lysate (∼10^10^–10^12^ PFU/ml) was used. The clearing zone or plaques in the spotted area indicated that the phage infected the tested strain. Each test was done in duplicate. A positive spot test was confirmed by plaque assay.

### Electron Microscopy

Each ultracentrifuge-purified high-titer phage was negatively stained with 2% (w/v) aqueous uranyl acetate (pH 4) on carbon-coated grids and examined with a transmission electron microscope (JEM 1200EX TEM, JEOL) at an accelerating voltage of 80 kV. Electron micrographs were taken at a magnification of × 50,000 in the Electron Microscopy Center, NCSU.

### Phage Structural Proteins

The structural proteins of phages were analyzed using the method previously described by Lu et al. (2003) with some modifications. Briefly, an ultracentrifuge-purified phage was mixed with SDS-PAGE sample buffer and then heated in a boiling water bath for 10 min. The boiled sample was allowed to cool to room temperature before loading onto a NuPAGE precast gradient minigel (4–12% Bis-Tris, Invitrogen Corporation, Carlsbad, CA, United States). Electrophoresis was carried out at 75 V for 2 h. The gel was stained with SimplyBlue SafeStain (Invitrogen). Novex^®^ Sharp Pre-stained Protein Standard (Invitrogen) was used to estimate the molecular weights of the separated phage proteins.

### Phage DNA Extraction and Restriction Analysis

Genomic DNAs of the selected phages were extracted from highly concentrated phage lysates using the phenol-chloroform extraction method as described by Lu et al. (2003) and digested with restriction endonucleases (*Eco*RI and *Hin*dIII; New England BioLabs, Beverly, MA, United States) according to the manufacturer’s recommendations. The resulting DNA fragments were separated on the 1% agarose gel containing 0.001% SYBR Safe DNA gel stain (Invitrogen) by gel electrophoresis in Tris-borate-EDTA buffer at 70 V (constant voltage) for 2 h. The 1 kb DNA ladder (Promega, Madison, WI, United States) was used to estimate the size of the DNA fragments.

### One-Step Growth Kinetics

The one-step growth curve of a selected phage was measured at the initial multiplicity of infection (MOI) of 0.01 in CJ. The infection tube containing phage (approximately 1 × 10^5^ PFU/ml), host culture (1 × 10^7^ PFU/ml), and CJ was incubated in a water bath at 37°C for 20 min (to allow for phage adsorption) and then centrifuged at 16,110 × *g* for 1 min. The supernatant (which contained un-adsorbed phage particles) was discarded. The remaining pellet was washed once with CJ and then re-suspended in CJ. The suspension was incubated in a water bath at 37°C. Samples were taken at 10-min intervals over a period of 100 min. Each sample was immediately diluted and subjected to plaque assay. All assays were carried out in duplicate. The experiment was independently repeated three times. The latent period was defined as the time interval between the end of the adsorption and the beginning of the first burst, as indicated by the initial rise in phage titer (Ellis and Delbruck, 1939; Adams, 1959). Burst size was calculated as the ratio of the final titer of liberated phage particles to the initial concentration of infected bacterial cells during the latent period (Adams, 1959).

### Thermal Stabilities

The thermal stabilities of phage ΦEnt and host *Salmonella* Thompson were independently evaluated in saline (0.85% NaCl) and TSB at 37, 50, 63, and 72°C. The initial phage titer and host concentrations were approximately 10^6^ PFU/ml and 10^6^ CFU/ml, respectively. A set of 1.5-ml tubes containing 900 μl of saline or TSB were pre-heated in a water bath to reach the test temperature. Then 100 μl of phage or host solution was added to a pre-heated tube. After briefly vortexed, the tube was immediately placed back into the water bath and heated for the indicated time periods. A sample (100 μl) was taken from this tube and immediately transferred into a tube containing 900 μl of saline at room temperature. The original tube was discarded. A new preheated tube was used for another treatment at the same or different temperatures following the same procedure. The titer of the surviving phages and the concentration of the surviving cells were measured by plaque assay and plate count method, respectively. Each sample was plated in duplicate. All experiments were repeated at least three times.

### Efficacy of Phage Infection Against *Salmonella* in Model Food Systems

The lytic activity of phage ΦEnt infection to control *S*. Thompson was evaluated at three different MOIs in CJ and BB as model food systems. The frozen CJ was thawed at 4°C and then equilibrated to room temperature before use. An overnight host culture was diluted with CJ to a concentration of ca. 10^5^ CFU/ml. A 9.9 ml aliquot of the CJ-diluted bacterial culture was added to each of the four 15-ml tubes. To one of the three tubes, 100 μl of phage stock at a specific titer was added to achieve an initial MOI of 1, 10, or 100. To the fourth tube, 100 μl of saline was added. This tube without phage served as a control. After mixing briefly, the four tubes were incubated statically in a water bath at 37°C. Hourly samples were taken from each tube for a 5-h period. Each sample was then serially diluted and plated onto a TSA plate in duplicate. After incubation at 37°C overnight, each plate was examined to obtain a plate count for the calculation of cell concentration. The same procedure was followed when using BB as a model food system. Each experiment was independently conducted three times.

### Statistical Analysis

All experiments were carried out in triplicate, and each sample was plated in duplicate. One-way analysis of variance (ANOVA) was performed using Statistica for Windows (StatSoft, Tulsa, OK, United States). Tukey’s Honest Significant Difference test was used to compare the mean values of data for significant difference (*P* ≤ 0.05).

## Results and Discussion

### Phage Isolation

A total of 14 *Salmonella* phages were isolated using 12 common *Salmonella enterica* serovars and/or subspecies as potential target hosts for phage enrichment and isolation (Table 1). Nine phages were isolated from turkey ceca (Tc), while the remaining five were isolated from turkey feces (Tf). Ten of the potential target hosts were susceptible to at least one isolated phage. No phage was isolated against two *S*. Typhimurium strains (B0038 and ATCC 14028). Six phages (Φ205, Φ206, Φ207, ΦEnt, ΦMont, and Φ13314) produced clear and relatively large plaques (1.3–3 mm in diameter), while the other eight phages produced clear but very small (<1 mm in diameter) or turbid plaques (data not shown). Turbid plaque on a host lawn resulted from incomplete lysis, which may indicate potential lysogeny as the induction of a prophage inside bacterial strains could lead to turbid plaques, which is often a characteristic of temperate phages (Campoy et al., 2006; Gallet et al., 2011; Thanki et al., 2019). Turbid clearing may also indicate partial resistance of the bacterial host to the phage, thus only a sub-population of cells was lysed (Bull et al., 2014). Phages that produce turbid plaques, therefore, may not be good candidates for use as biocontrol agents (Chan et al., 2013; Abedon et al., 2017). Thus, only the six phages that produce clear plaques with a diameter > 1 mm were selected for characterization.

### Host Range

The host ranges of six selected phages were determined using spot tests against a panel of 22 *Salmonella* strains comprising diverse serovars that were commonly associated with the foodborne Salmonellosis and 10 other Gram-negative bacteria including seven *Shigella* strains, two *Escherichia* coli strains, and one *Enterobacter cloacae* strain (Table 2). The six phages displayed different host ranges, indicating that they are distinct from one another. Host range is largely dependent on the ability of phages to bind to either a single receptor (i.e., narrow host range) or multiple different receptors (i.e., broad host range; Fong et al., 2021). Φ206, ΦMont, and Φ13314 showed very narrow host ranges, infecting only one or two tested *Salmonella* strains. In contrast, Φ205, Φ207, and ΦEnt exhibited broader host ranges. Φ205 and Φ207 infect five and eight tested *Salmonella* strains, respectively. In addition, they both crossed the boundary of the genus and infected *Shigella sonnei* type strain (Table 2). ΦEnt displayed the broadest and very unusual host range, infecting 11 of the 22 (50%) tested *Salmonella* strains from nine different serovars (Belem, Cerro, Enteritidis, Typhimurium, Kentucky, Infantis, Hadar, Thompson, and Braenderup). Moreover, ΦEnt also broke the boundary of the genus and lysed three *Shigella* strains from two species (*S. dysenteriae* and *S*. *sonnei*). It is well-known that *S*. Enteritidis, Typhimurium, Kentucky, Infantis, Hadar, and Thompson belong to the most common and highly pathogenic *Salmonella* serovars (Anonymous, 2021), while *Shigella dysenteriae* and *S. sonnei* are important human pathogens causing bacterial dysentery and shigellosis (CDC, 2022). It is noted that ΦEnt and Φ207 (both siphophages) share seven *Salmonella* hosts from different serovars and one *Shigella* host (Table 2), which is not very common. The similarity in host ranges may indicate that these phages recognize similar receptors in the hosts (Kalatzis et al., 2016). Phages are generally species-specific or genus-specific (Hyman and Abedon, 2010; Cazares et al., 2021). Broad-host range *Salmonella* phages have been reported in the literature (Santos et al., 2011; Li, M. et al., 2020; Tao et al., 2021), but phages infecting bacteria from different genera are comparatively rare. It has been reported that phages SFP10 and KIT03 broke the boundary between *Salmonella* and *Escherichia* and infected both *Salmonella enterica* and *E. coli* O157:H7 (Park et al., 2012; Pham-Khanh et al., 2019). A few studies have reported the phages that can cross the genus boundary between *Salmonella* and *Shigella*. Hamdi et al. (2017) reported that phage SH7 was able to lyse strains of *Salmonella paratyphi* and *Shigella dysenteriae* (Hamdi et al., 2017). It is of interest to explore the genetic basis for ΦEnt and Φ207 to share similar host ranges and their ability to infect bacteria from two genera. Host range is critical in the selection of phage candidates for biocontrol agents and detection applications. To be considered as excellent candidates for use as biocontrol agents in foods, phages should be strictly lytic and possibly have a broad host range (Sharma et al., 2017; Fong et al., 2021; Picozzi et al., 2021). Since ΦEnt exhibited the broadest and unusual host range, formed clear plaques (possibly with high lytic activity), and had higher efficiency of plating (EOP) (data not shown), it was selected for further characterization of (with respect to) its growth kinetics, restriction pattern, and lytic profiles in model food systems and for determining its thermal stability.

### Electron Microscopy

**FIGURE 1:**
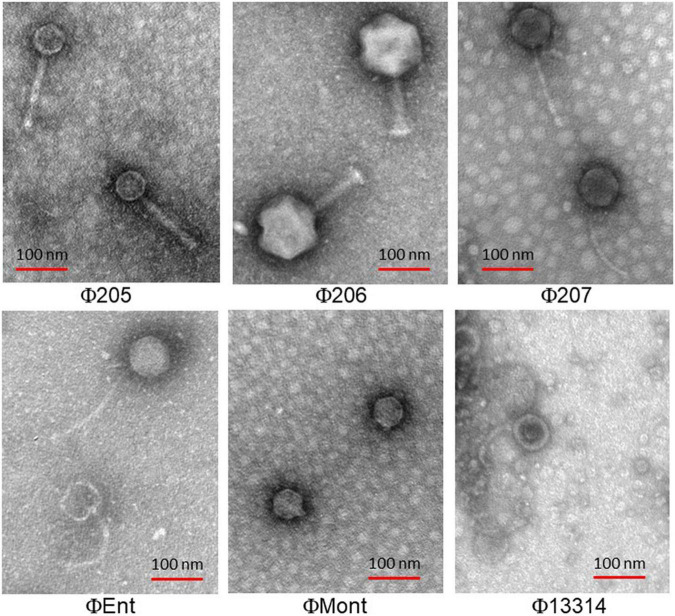
Transmission electron micrographs of six *Salmonella* phages isolated from turkeys.

The electron micrographs show that the six selected phages are all tailed phages with icosahedral heads and thus belong to the order *Caudovirales* (Figure 1). Three phages (Φ207, ΦEnt, and Φ13314) have long flexible, non-contractile tails and thus belong to the *Siphoviridae* family. Two phages (Φ205 and Φ206) have contractile tails and belong to the *Myoviridae* family. One phage (ΦMont) has a very short tail and belongs to the *Podoviridae* family.

### Structural Protein Profiles

**FIGURE 2:**
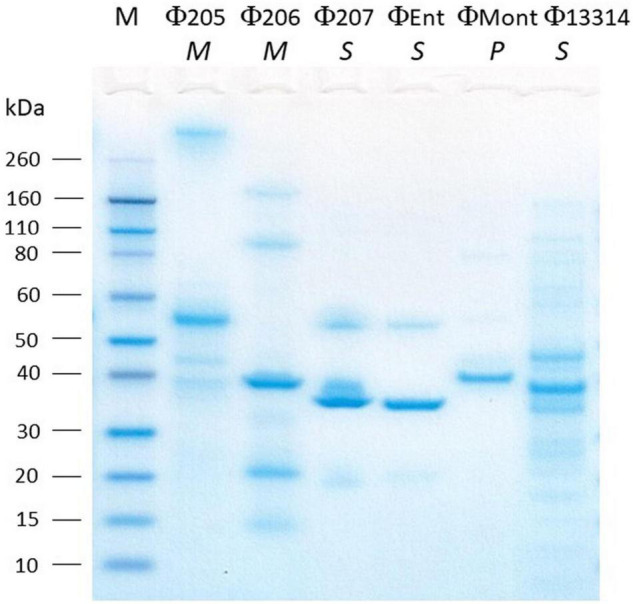
SDS-PAGE analysis of the structural proteins of six *Salmonella* phages isolated from turkeys. Lane M, molecular mass standard. The italicized letters (*M*, *S*, and *P*) below phage names stand for *Myoviridae*, *Siphoviridae*, and *Podoviridae*, respectively.

The SDS-PAGE analysis showed the structural protein profiles of the six *Salmonella* phages (Figure 2). Each phage showed at least one thick band representing the major structural proteins in the range of 30–60 kDa. However, each phage has a unique structural protein banding pattern, indicating that these phages are distinct from one another. Among the six phages, Φ13314 (a *siphoviridae* phage) showed the most bands (at least 12 bands). Φ207 and ΦEnt are also *siphoviridae* phages, but they showed much fewer bands than Φ13314. These two broadest host-range phages appeared to share two structural protein bands: one thick band (major structural protein) and a band between 50 and 60 kDa. Genetic analysis of the two phage genomes is needed to see if these two proteins are involved in phage attachment to the hosts, which might explain why these two phages shared seven *Salmonella* hosts. Φ205 and Φ206 (*Myoviridae* phages) showed 5–6 bands, while ΦMont (a *Podoviridae* phage) showed a few very faint bands besides a thick band. Lu et al. (2020) previously isolated eight phages and their Gram-negative bacterial hosts from a commercial cucumber fermentation. They demonstrated that the phages from the same phage family exhibited similar structural protein banding patterns. However, that was not the case with the six phages in this study.

### DNA Restriction Analysis of Phages Φ207 and ΦEnt Sharing Eight Hosts

**FIGURE 3:**
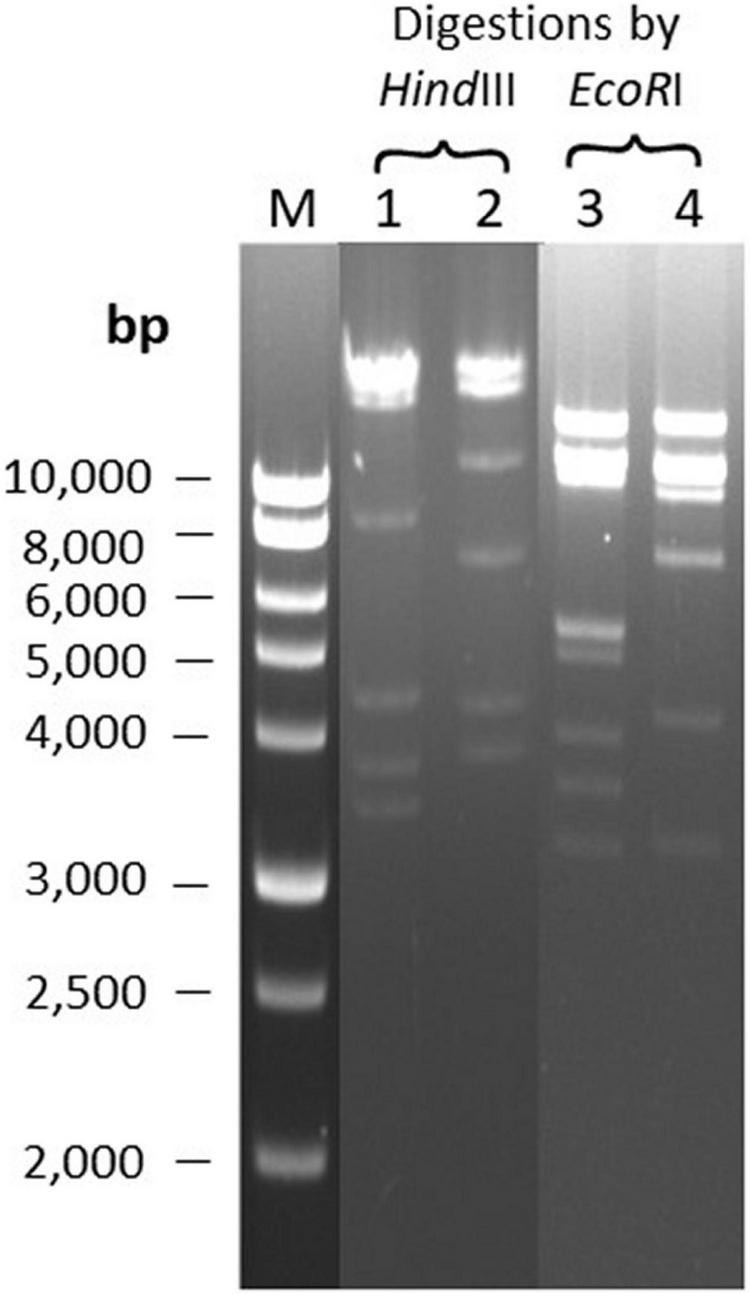
Restriction banding patterns of phage DNAs upon digestion by endonuclease *Eco*RI and *Hin*dIII. Lane M: 1 kb ladder; Lanes 1 and 3: digestion of Φ207 DNA; Lanes 2 and 4: digestion of ΦEnt DNA.

The two broadest host-range phages, Φ207, and ΦEnt (both siphophages), share seven different *Salmonella enterica* serovars and one *Shigella sonnei* strain (Table 2), which is rare. It is of interest to see if their genomes may share some common features. The two phage genomes were digested with two restriction endonucleases, *Eco*RI and *Hin*dIII. Figure 3 showed that both phage genomes can be digested by *Eco*RI and *Hin*dIII and generate multiple bands on the resolved gel, confirming that the phage genomes are dsDNA. Φ207 and ΦEnt showed different restriction banding patterns from *Eco*RI digestion or from *Hin*dIII digestion, confirming the unique identities of these phages. Nevertheless, they shared three restriction bands (two bands with molecular weights above 10,000 bp and one band with molecular weight of approximately 3,200 bp) from *Eco*RI digestion, suggesting the conservation of some *Eco*RI-specific cutting sites. Although siphoviruses are known to exhibit remarkable genomic mosaicism (Santander et al., 2017), it is not surprising that phages from the same family share some components and thus exhibit some notable degree of similarity among siphophages (Fong et al., 2017). This may partially explain why Φ207 and ΦEnt (both siphophages) showed similar, but not identical, broad host ranges. Santander et al. (2017) reported that *Salmonella* phages fSE1C and fSE4C showed very similar banding patterns from *Eco*RI or *Hin*dIII digestion. They found that the two phage genomes shared 43% sequence similarity, with genes involved in structure, replication, host specificity, and DNA metabolism indicating remarkable conservation. Complete genome analyses of Φ207 and ΦEnt are needed to reveal the genetic basis for the two phages sharing seven *Salmonella* hosts and one *Shigella* host and for their relevance for practical application.

### One-Step Growth Kinetics of Phage ΦEnt

**FIGURE 4:**
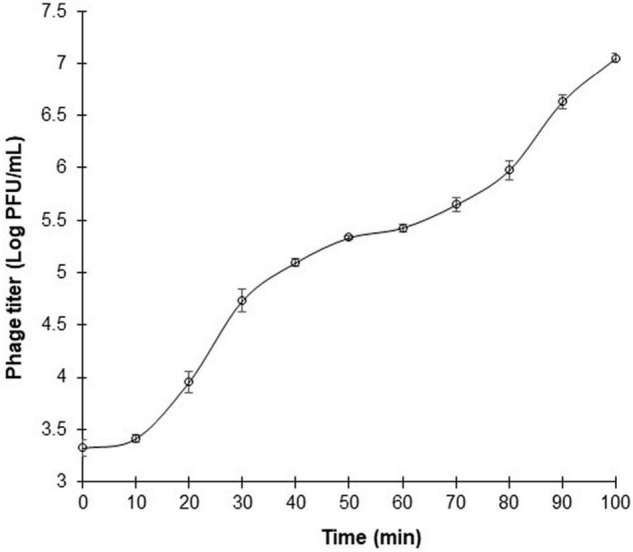
One-step growth curve of phage ΦEnt infecting *Salmonella* Thompson at MOI 0.01 in cucumber juice at 37°C. The latent period is approximately 10 min, and the burst size is about 100 phage particles per infected cell. Error bars indicate the standard error of the mean.

One-step growth curve of phage ΦEnt was measured in CJ (as a model food system) at MOI of 0.01 against *S*. Thompson instead of *S*. Enteritidis (the target host used for phage isolation) because ΦEnt formed larger clear plaques on the lawn of *S*. Thompson, suggesting that ΦEnt has a higher efficacy against *S*. Thompson than the primary strain of isolation. Figure 4 shows that ΦEnt has a short latent period of 10 min, which is shorter (25–65 min) than that of many other reported *Salmonella* phages (Huang et al., 2018a; El-Dougdoug et al., 2019; Duc et al., 2020). A shorter latent period has been positively related to the lytic activity of the phage (Li, Z. et al., 2020). ΦEnt also exhibited a relatively large burst size (∼100 PFU/infected cell). For many *Siphoviridae* and *Myoviridae* phages, the typical burst size ranged es, the typical burst size raes, the typical burst size ra 50 and 100 PFU/infected cell (Park et al., 2012; Litt and Jaroni, 2017; Khalatbari-Limaki et al., 2020). A phage with both a short latent period (15 min or less) and a large burst size (> 50 PFU/cell) may have a selective advantage over competing phages, resulting in very high lytic activity (Park et al., 2012). Thus, such a phage is desirable for use in the food industry (Kalatzis et al., 2016).

### Thermal Stabilities of Phage ΦEnt and Host *Salmonella* Thompson

**FIGURE 5:**
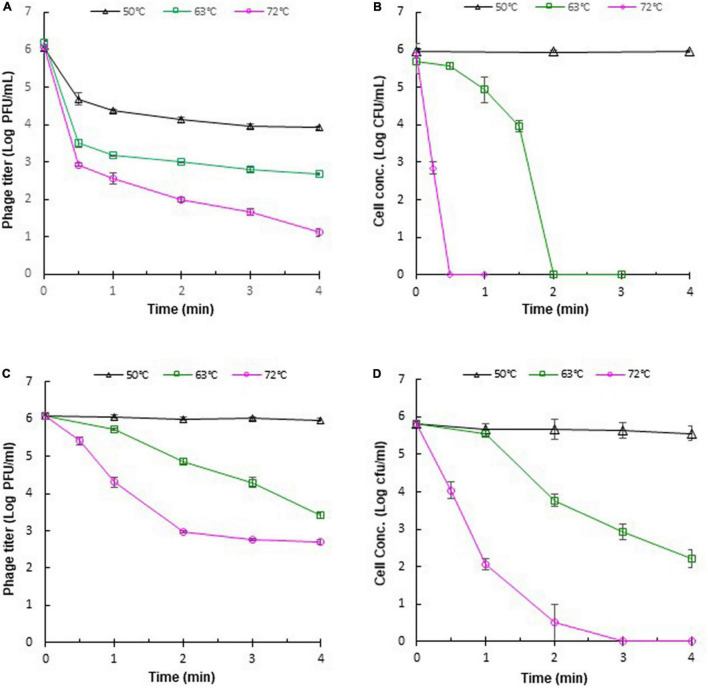
Thermal stabilities of *Salmonella* phage ΦEnt and its host *Salmonella* Thompson in saline (**A,B**, respectively) and in TSB (**C,D**, respectively). Error bars indicate the standard error of the mean.

The stability of phages against adverse effects during food processing is important for phage application. Heat treatments, such as pasteurization, are often applied in the food industry. Therefore, it is necessary to evaluate the thermal stabilities of phages for their application under practical conditions. The thermal stabilities of phage ΦEnt and host *S.* Thompson were evaluated in saline (0.85% NaCl) at 37, 50, 63, and 72°C. It was found that both ΦEnt and the host were stable at 37°C (data not shown), but at higher temperatures, ΦEnt and the host exhibited different trends in thermal stability. During the first 30 s at 50, 63, and 72°C, phage titer declined rapidly, resulting in 1. 5-, 2. 5-, and 3-log_10_ reduction, respectively (Figure 5A). But after 30 s at these temperatures, the decline in ΦEnt titer was much slower. Exposure to 50, 63, and 72°C for 4 min resulted in 2-, 3. 3-, and 5-log_10_ reduction in ΦEnt titers, respectively. Significant numbers of the phage (10^4^, 10^3^, and 10 PFU/ml) survived the 4-min heating at 50, 63, and 72°C, respectively. In contrast, the host was stable at 50°C during the 4-min heating (Figure 5B). At 63°C, host concentration initially decreased slowly during the first 30 s, but rapidly thereafter. It only took 2 min at 63°C or 30 s at 72°C to completely inactivate all the host cells (6-log_10_ reduction). These results demonstrated that the host in saline was much more sensitive to 63 or 72°C than the phage. The thermal stabilities of ΦEnt and the host were further evaluated in the TSB medium. It was found that the phage titer and cell concentration remained unchanged at 37°C (data not shown) and 50°C (Figures 5C,D), indicating that both the phage and its host were very stable in TSB at these temperatures. As the temperature increased, their stabilities decreased, but at different rates. Exposure to 63 and 72°C for 4 min in TSB resulted in 2.6-log_10_ and 3.3-log_10_ unit reductions in ΦEnt titer, respectively. These reductions were much smaller than those (3.3-log_10_ at 63°C and 5-log_10_ at 72°C) observed in saline, demonstrating that ΦEnt had significantly (*p* < 0.05) better thermal stability in TSB than in saline. It should be noted that the exposure to 72°C for 15 s (the typical pasteurization condition used in food industries) caused less than 0.6-log_10_ unit reduction in phage titer (Figure 5C), indicating that ΦEnt retained the good stability required for use as biocontrol agent under commonly encountered pasteurization conditions. The host also showed better stability at 63 and 72°C in TSB (Figure 5D) than in saline (Figure 5B). However, compared with ΦEnt in TSB (Figure 5C), the host was significantly (*p* < 0.05) less stable at 63 and 72°C (Figure 5D). Exposure to 63°C for 4 min caused a 3.6-log_10_ reduction in host concentration in TSB (1-log_10_ more reduction compared to that for phage). At 72°C, the host was completely eliminated within 3 min, while many phage particles (5 × 10^2^ PFU/ml) survived the 4-min heating.

The differences in the thermal stabilities of the phage or the host in TSB and saline could be attributed to the components other than salt (such as peptides and proteins) in TSB, which provided thermal protection to ΦEnt and the host. Several other studies also noted that the medium components of phage solution could affect the thermal stability of phages (Fabrizio et al., 1999; Sua’/rez and Reinheimer, 2002; Quiberoni et al., 2003; Briggiler Marco et al., 2009). It was reported that reconstituted non-fat dry skim milk is more protective than De Man, Rogosa and Sharpe (MRS) broth against thermal inactivation of some dairy phages (Daoust et al., 1965; Quiberoni et al., 2003). Whole milk provides better thermal protection than skim milk for *Lactococcus lactis* phages (Fabrizio et al., 1999). Tris-magnesium gelatin buffer was more effective in protecting *L. lactis* phages against heat exposures than M17 broth (Sua’/rez and Reinheimer, 2002). It can be expected that different foods provide different levels of thermal protection to phages or other organisms. The thermal stability of phages may depend on their DNA content (Yamagishi and Ozeki, 1972). It was reported that *Listeria* phage A511 with a denser head capsid content showed higher thermal stability than *Listeria* phage P100 (Ahmadi et al., 2017). Heating could cause irreversible damage to phage particles, such as irreversible unfolding and dissociation of structural proteins, damage to the portal complex, disintegration of phage into head and tail, and leakage of DNA (Ahmadi et al., 2017). Shang et al. (2021) visualized the heat-treated phages with a transmission electron microscope. The detrimental effects of heat on phage structure were evidenced by phage tail aggregation, detachment of phage head and tail, and empty capsid.

### Efficacy of Phage ΦEnt Infection Against *Salmonella* in Model Food Systems

**FIGURE 6:**
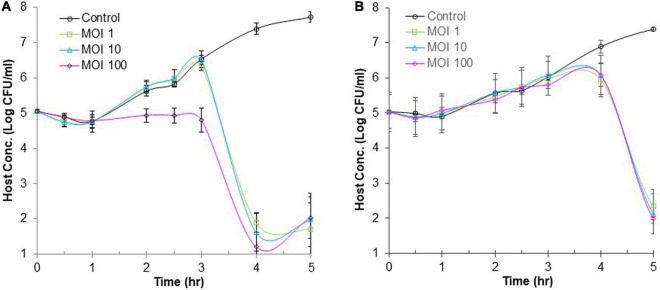
Efficacy of phage infection by ΦEnt against *S.* Thompson in cucumber juice **(A)** and beef broth **(B)**. Each point represents the mean ± standard error based on three measurements.

Many foodborne outbreaks of salmonellosis were linked to animal-based foods and vegetables. Thus, BB (simulating meats) and CJ (simulating vegetables) were used as model food systems in this study to evaluate the efficacy of phage ΦEnt infection against *S*. Thompson at three different MOIs (1, 10, and 100). Figure 6A shows changes in the host concentration in the control and infection tubes containing CJ during 6 h. During the first hour of incubation, the host concentrations in all tubes (control and infection tubes) were the same (with a slight decrease due to the medium change from TSB to CJ). But during the following 2 h (hours 2 and 3), the host concentration in the control tube and the infection tubes with initial an MOI of 1 or 10 increased rapidly at the same rate, while the host concentration in the infection tube with initial MOI of 100 remained unchanged. During the fourth hour of incubation, the host concentration in the control tube continued to increase rapidly and reached 2.4 × 10^7^ CFU/mL. In contrast, the host concentration in all the three infection tubes dramatically decreased, resulting in ≥ 4-log_10_ unit reduction in host concentration (causing host concentration < 10^2^ CFU/mL, Figure 5). At the end of the fifth hour, the final host concentrations in the control tube increased to 5.3 × 10^7^ CFU/mL, while the final host concentration in all three infection tubes (regardless of MOI) was ≤ 10^2^ CFU/mL. Such a large difference (more than 5-log_10_ units) in final host concentration between the control and the infection tubes indicated that ΦEnt infection is highly effective in inhibiting and even eliminating the *Salmonella* host in CJ. The results also indicated that given enough time (4 h) the phage infection with an initial MOI of 1 could achieve the same log reduction in host concentration as those with higher initial MOIs (10 and 100). Huang et al. (2018b) evaluated the efficacy of the phage infection by a *Salmonella* phage LPSE1 against *S*. Enteritidis ATCC 13076 on lettuce leaves and found that 5-h phage infection at an initial MOI of 1, 10, or 100 resulted in only 2-log_10_ reduction. Several other studies reported that *Salmonella* phage infection resulted in a 1.7-log_10_ reduction in host concentration in lettuce (Spricigo et al., 2013), 3-log_10_ reduction in Chinese cabbage (Bao et al., 2015), 1.37-log_10_ reduction in mustard, and 0.55-log_10_ reduction on broccoli (Pao et al., 2006). None of these studies achieved the same level (≥ 4-log_10_) of the reduction as achieved in CJ in our study. This is probably partially due to the fact that the solid food matrices limit mass transport/diffusion and reduce phage–host interaction (Whichard et al., 2003; Hudson et al., 2010). Figure 6A reveals that the increase in cell concentration occurred in all the three infection tubes at 5 h of incubation, indicating that phage resistance mutants emerged. Bacteria can become resistant to phages through different mechanisms, such as spontaneous mutations in bacterial cell surface receptors, restriction modification systems, and adaptive immunity *via* the CRISPR-Cas system (Labrie et al., 2010; Mangalea and Duerkop, 2020). It was observed that some surviving host cells formed much smaller and/or irregularly shaped colonies after 3 h of infection (data not shown), indicating a cytopathic effect from phage infection.

Figure 6B shows changes in the host concentration in the control and infection tubes containing BB during 6 h. The host concentration in all tubes (regardless of MOI used) increased approximately by 1-log unit during the first 3.5 h of incubation. Thereafter, the host concentration in the control continued to increase and reached 3.7 × 10^7^ CFU/mL at the end of the sixth hour. In contrast, the host concentration in the infection tubes (regardless of MOI) decreased rapidly after 3.5 h of treatment. At 6 h, the cell concentration in infection tubes was either below or slightly above 10^2^ CFU/ml, indicating a 6-log_10_ reduction in host concentration compared to the controls. These results demonstrated that the 6-h ΦEnt infection at all MOIs tested is highly effective to suppress and even eliminate the host in BB. Huang et al. (2018b) assessed the anti-*Salmonella* efficacy of phage LPSE1 in pork sausage at 28°C and found that 6-h phage infection at MOIs of 1 and 100 only resulted in 2-log_10_ and 2.6-log_10_ reduction in host concentration, respectively, which were much lower than those achieved by ΦEnt in BB in our study. Such a big difference may be partially explained by the fact that BB as a liquid matrix provides better homogeneity and an opportunity for a phage to come in contact with its host. It is expected that higher MOI and longer incubation time may be needed to achieve the same log reduction in cell concentration if using solid food matrices. Shang et al. (2021) evaluated the ability of phage vB_SalP_TR2 to reduce *Salmonella* Albany contamination in pasteurized milk and chicken meat stored at 37°C for 6 h. They found that the phage was much more effective against *S*. Albany in pasteurized milk than in chicken meat (Shang et al., 2021).

The high efficacy of ΦEnt against *S*. Thompson observed in both model food systems is largely due to its short latent period (10 min) and large burst size (100 PFU/cell). The evaluation of the efficacies of ΦEnt against multiple other susceptible *Salmonella* serovars is underway. It is of great interest in evaluating the efficacy of ΦEnt against the three susceptible *Shigella* strains to further explore its biocontrol potential.

## Conclusion

In conclusion, this study isolated and characterized the phages from turkeys against various *Salmonella* serovars that were commonly associated with foodborne Salmonellosis. Six selected phages were from all three families in the *Caudovirales* order. Each of them had a unique host range and a structural protein profile. The two broadest host range phages shared seven different *Salmonella* serovars and one *Shigella sonnei* strain. ΦEnt showed a remarkable and very unusual broad host range (infecting 11 *Salmonella* strains in nine serovars and three *Shigella* strains in two species, *dysenteriae* and *sonnei*), high lytic activity at all MOIs tested against *Salmonella* in two model food systems, and good thermal stability under pasteurization conditions. These desirable characteristics suggested that ΦEnt is a novel phage with great potential to be used as an effective biocontrol agent to control *Salmonella* in foods. Further studies are needed to assess the efficacy of ΦEnt against three susceptible *Shigella* strains to further explore its biocontrol potential. It would be also needed to evaluate ΦEnt and other isolated phages against multiple *Salmonella* serovars in various food matrices. Additionally, comparative genomic analysis of these phages, particularly the two broad host range phages, needs to be conducted to explore the genetic basis for phage–host interactions.

## Data Availability Statement

The original contributions presented in this study are included in the article/supplementary material, further inquiries can be directed to the corresponding author.

## Author Contributions

ZL contributed to the conceptualization, funding acquisition, experimental design, phage isolation and purification, all other experiments, data analysis, draft manuscript, and manuscript revision. JM, ST, HM, DI, SKo, RD, HT, RP, and AR contributed to the experiments. FB contributed to electron microscopy analysis and manuscript revision. SKa contributed to sample resources and manuscript revision. All authors contributed to the article and approved the submitted version.

## Conflict of Interest

The authors declare that the research was conducted in the absence of any commercial or financial relationships that could be construed as a potential conflict of interest.

## Publisher’s Note

All claims expressed in this article are solely those of the authors and do not necessarily represent those of their affiliated organizations, or those of the publisher, the editors and the reviewers. Any product that may be evaluated in this article, or claim that may be made by its manufacturer, is not guaranteed or endorsed by the publisher.
